# High-Dimensional Mediation Analysis Based on Additive Hazards Model for Survival Data

**DOI:** 10.3389/fgene.2021.771932

**Published:** 2021-12-23

**Authors:** Yidan Cui, Chengwen Luo, Linghao Luo, Zhangsheng Yu

**Affiliations:** ^1^ Department of Bioinformatics and Biostatistics, School of Life Sciences and Biotechnology, Shanghai Jiao Tong University, Shanghai, China; ^2^ SJTU-Yale Joint Center for Biostatistics, Shanghai Jiao Tong University, Shanghai, China; ^3^ Public Laboratory, Taizhou Hospital of Zhejiang Province, Wenzhou Medical University, Linhai, Zhejiang, China; ^4^ Clinical Research Institute, Shanghai Jiao Tong University School of Medicine, Shanghai, China

**Keywords:** high-dimensional mediators, additive hazards model, survival data, mediation analysis, SIS

## Abstract

Mediation analysis has been extensively used to identify potential pathways between exposure and outcome. However, the analytical methods of high-dimensional mediation analysis for survival data are still yet to be promoted, especially for non-Cox model approaches. We propose a procedure including “two-step” variable selection and indirect effect estimation for the additive hazards model with high-dimensional mediators. We first apply sure independence screening and smoothly clipped absolute deviation regularization to select mediators. Then we use the Sobel test and the BH method for indirect effect hypothesis testing. Simulation results demonstrate its good performance with a higher true-positive rate and accuracy, as well as a lower false-positive rate. We apply the proposed procedure to analyze DNA methylation markers mediating smoking and survival time of lung cancer patients in a TCGA (The Cancer Genome Atlas) cohort study. The real data application identifies four mediate CpGs, three of which are newly found.

## 1 Introduction

Lung cancer continues to be the most common cancer type worldwide with the highest (18%) death rate among all malignant tumors (Wild et al., 2020). Zeilinger et al. (2013) found that tobacco smoking has an extensive genome-wide influence on DNA methylation. Meanwhile, Tsou et al. (2002) discovered that DNA methylation has a strong relationship with lung cancer. It is of interest to study how DNA methylation mediates the causal pathway between smoking and lung cancer patient’s survival.

Mediation analysis, for potential indirect effects (IEs) detection, was first applied to psychological theory and research (Baron and Kenny, 1986). Then this idea was generally applied to sociological and biomedical fields (Kahler et al., 2017; Lapointe-Shaw et al., 2018; Vansteelandt et al., 2019; Arora et al., 2020; Song et al., 2020). The mediation model can be expressed in the following equations:

Y=c+γX+ε (1)

$$M=c_{m}+\alpha X+\varepsilon$$ (2)

$$Y_{M}=c_{y}+\gamma^{\prime }X+\beta M+\varepsilon ,$$ (3)

where *Y* and *Y*_*M*_ are the outcomes, *M* is the mediator, and *X* is the exposure. Eq. 1 is the original regression model. Eq. 2 models the *X*’s effect on *M*, and Eq. 3 models the *X*’s effect on *Y* adjusting for *M*. Estimation and inference of IE are essential to mediation analysis, which includes (MacKinnon et al., 2002) the causal steps tests (Judd and Kenny, 1981; Baron and Kenny, 1986), the coefficients difference tests (Freedman and Schatzkin, 1992), and the coefficients product tests (Sobel, 1987). Mediation analysis has been extended from univariate to multivariate or even high-dimensional mediators. Meanwhile, the outcome could be continuous, binary, longitudinal data (Selig and Preacher, 2009), as well as survival data (VanderWeele, 2011).

While the Cox model serves a purpose to survival data analysis, the additive hazards model becomes more and more common now, which could model the time-varying effect directly (Aalen, 1989). Lin and Ying (1994) studied a semiparametric method by mimicking the Cox proportional hazards model estimation method. Yin and Cai (2004) proposed an estimated method for multivariate failure time data and demonstrated its convergence properties. Mediation analysis has been applied to the additive hazards model. The early study for natural IE estimation was presented by Lange and Hansen (2011). Then, the study has been extended to multiple mediators (Taylor et al., 2008; Huang and Yang, 2017), and time-dependent mediators (Deboeck and Preacher, 2016; Aalen et al., 2020).

In recent years, scientists utilized the additive hazards model to analyze high-dimensional time-to-event data. Lin and Lv (2013) compared five penalized regularization methods and found that SCAD (smoothly clipped absolute deviation (Fan and Li, 2001)), MCP (minimax concave penalty (Zhang, 2010)), and SICA (smooth integration of counting and absolute deviation (Lv and Fan, 2009)) have better performance. Chen et al. (2019) proposed a screening method based on a sparsity-restricted pseudo-score estimator for ultrahigh-dimensional sparse data with an additive hazards model. On the other hand, extensive works have been done in high-dimensional mediation analysis. Zhang et al. (2016) applied high-dimensional mediation analysis to investigate DNA methylation sites mediating the causal pathway from smoking to reduced lung function. Latent variables, Cox model, nonlinear mediators, and sparse PCA are also discussed for high-dimensional mediation analysis (Derkach et al., 2019; Loh et al., 2020; Luo et al., 2020; Zhao et al., 2020), as well as IE testing methods (Djordjilović et al., 2019; Gao et al., 2019; Dai et al., 2020; Liu et al., 2021).

However, the analytical approach for high-dimensional mediators based on the additive hazards model is still lacking. We aim to establish a procedure for additive hazards model and investigate DNA methylation markers with IE between tobacco smoking and lung cancer patient’s survival. The main idea of the proposed procedure is to reduce high-dimensional mediators by the “two-step” sure independence screening (SIS)–SCAD method and identify positive mediators by the Sobel test. We apply SIS in the first step for its oracle property and large-scale dimensionality reduction studied by Fan and Lv (2008), who also demonstrated that combining SIS and SCAD can perform the variable selection and parameter estimation simultaneously. SIS has been extended to survival analysis with Cox proportional data (Zhao and Li, 2012) and additive hazards model (Gorst-Rasmussen and Scheike, 2013). We apply the SCAD penalty in the second step with the utilization of the R package “haza” (Gorst-Rasmussen and Scheike, 2012).

The rest of this article proceeds as follows. In the next part, we present methodological materials involving notations, assumptions, and detailed procedures. Then, we provide simulation studies to evaluate the proposed procedure’s performance and a factual data application to identify mediate CpGs between smoking and lung cancer patients’ survival time. Conclusion and discussion are then included at last.

## 2 Materials and Methods

### 2.1 Notation and Models of the Proposed Procedure

For each individual *i* = 1, 2, *…*, *n*, *T*_*i*_ = min(*D*_*i*_, *C*_*i*_) denotes the observed survival time, where *D*_*i*_ is the time from beginning to the event and *C*_*i*_ is the censoring time. *δ*_*i*_ = *I*(*D*_*i*_ ≤ *C*_*i*_) is the failure indicator, and *I*(⋅) is the indicator function. When *D*_*i*_ > *C*_*i*_, the participant is said to be right-censored, which we consider more in this article. Censoring rate, representing the rate of participants whose information is not available due to loss to follow-up or nonoccurrence of the interested event within the trial duration, is significant to survival analysis (Prinja et al., 2010).

**FIGURE 1:**
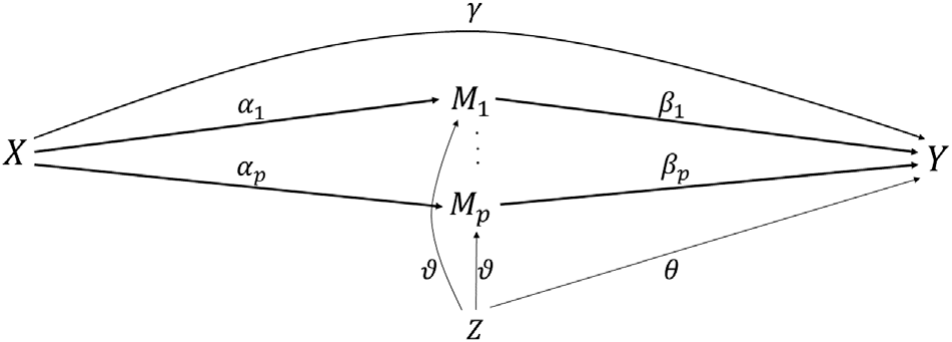
Direct acyclic graph with the exposure, outcome, and high-dimensional mediators.

Figure 1 is a direct acyclic graph showing the relationship between exposure, outcome, covariates, and high-dimensional mediators. ***X*** is the exposure. $M={\left\{M_{1},M_{2},\ldots ,M_{p}\right\}}^{T}$ denotes the high-dimensional mediators, and *p* ≫ *n*. ***Y*** is the survival outcome. ***Z*** represents covariates. The additive hazards model with mediators is:

$$\lambda_{i}\left(t|X_{i},M_{i},Z_{i}\right)=\lambda_{0i}\left(t\right)+\gamma X_{i}\left(t\right)+\theta^{T}Z_{i}\left(t\right)+\overset{p}{\underset{k=1}{\sum }}\beta_{k}M_{ki}\;i=1,2,\ldots ,n,$$ (4)

$$M_{ki}=c_{k}+\alpha_{k}X_{i}\left(t\right)+\vartheta^{T}Z_{i}\left(t\right)+e_{ki}\;k=1,2,\ldots ,p.$$ (5)

Eq. 4 is an additive hazards model showing individual’s hazard rates. *λ*_*i*_ is associated with exposure, covariates, and high-dimensional mediators. *λ*_0*i*_(*t*) indicates the time-varying intercept. Eq. 5 describes the way how exposure and covariates linearly influence mediators. *c*_*k*_ is the intercept and *e*_*ki*_ is random error.

### 2.2 Assumptions

To obtain a causal inference conclusion from the mediation analysis, we make some assumptions about mediators and confounders. Here, *T*(*x*, *M*_1_, *M*_2_, *…*, *M*_*p*_) denotes that the survival time depends on *X* and *M*_*k*_(*k* = 1, 2, *…*, *p*). *M*_*k*_(x*) represents the mediators with different exposure values. The consistency assumption matters to the proposed procedure requiring to hold the outcome once the exposure and mediators were set (VanderWeele and Vansteelandt, 2009; Rehkopf et al., 2016). Based on Luo et al. (2020) and Huang and Yang (2017), the assumptions for the proposed procedure are as follows:

1) *X* ⊥ *T*(*x*, *m*_1_, *m*_2_, *…*, *m*_*p*_)|*Z*; there is no unmeasured confounding effect between *X* and *T* conditional on *Z*.
2) For any *k* = 1, 2, *…*, *p*, *M*_*k*_ ⊥ *T*(*x*, *m*_1_, *m*_2_, *…*, *m*_*p*_)|*X*, *Z*; there is no unmeasured confounding effect between *M*_*k*_ and *T* conditional on *X* and *Z*.
3) For any *k* = 1, 2, *…*, *p*, *X* ⊥ *M*_*k*_|*Z*; there is no unmeasured confounding effect between *X* and *M*_*k*_ conditional on *Z*.
4) For any *k* = 1, 2, *…*, *p*, $M_{k}^{x*}⊥T\left(x,m_{1},m_{2},\ldots ,m_{p}\right)|Z$ ; there is no *X*-induced factor confounding the pathway from *M* to *T* conditional on *Z*, where x* is intervention for *X* with different value than *x*.

### 2.3 Proposed Procedure

Referring to the counting process notation, *N*_*i*_(*t*) = *I*(*T*_*i*_ ≤ *t*, *δ*_*i*_ = 1) represents the observed failure counting process, where *δ*_*i*_ = *I*(*D*_*i*_ ≤ *C*_*i*_). *Y*_*i*_(*t*) = *I*(*T*_*i*_ ≥ *t*) is the at-risk indicator. And

$$M_{i}\left(t\right)=N_{i}\left(t\right)-\int_{0}^{t}Y_{i}\left(s\right)\left\{\lambda_{0i}\left(s\right)+\gamma X_{i}\left(s\right)+\theta^{T}Z_{i}\left(s\right)+\beta^{T}M_{i}\left(s\right)\right\}ds$$

is the additive martingale process. Let ***P*** = (*γ*, *θ*, *β*) and ***Q***_*i*_ = (*X*_*i*_, *Z*_*i*_, *M*_*i*_). Then the martingale could be simplified as $M_{i}\left(t\right)=N_{i}\left(t\right)-\int_{0}^{t}Y_{i}\left(s\right)\left\{\lambda_{0i}\left(s\right)+P^{T}Q\right\}ds$ .

According to Lin and Ying (1994), the pseudo-likelihood score function of the proposed model is:

$$U\left(P\right)=\overset{n}{\underset{i=1}{\sum }}\int_{0}^{\infty }\left\{Q_{i}\left(t\right)-\bar{Q}\left(t\right)\right\}\left\{dN_{i}\left(t\right)-Y_{i}\left(t\right)P^{T}Q_{i}\left(t\right)dt\right\},$$

where $\bar{Q}\left(t\right)=\sum_{j=1}^{n}Y_{j}\left(t\right)Q_{j}\left(t\right)/\sum_{j=1}^{n}Y_{j}\left(t\right)$ . Referring to Lin and Lv (2013), we can write the score function into

$$U\left(P\right)=b-VP,$$

where

$$b=\frac{1}{n}\overset{n}{\underset{i=1}{\sum }}\int_{0}^{\infty }\left\{Q_{i}\left(t\right)-\bar{Q}\left(t\right)\right\}dN_{i}\left(t\right),\;V=\frac{1}{n}\overset{n}{\underset{i=1}{\sum }}\int_{0}^{\infty }Y_{i}\left(t\right){\left\{Q_{i}\left(t\right)-\bar{Q}\left(t\right)\right\}}^{⊗2}dt,$$

and ***a*** ^⊗2^ = ***aa*** ^*T*^. Then the least-squares type loss function of the proposed model is:

$$L\left(P\right)=\frac{1}{2}P^{T}VP-b^{T}P.$$ (6)

**FIGURE 2:**
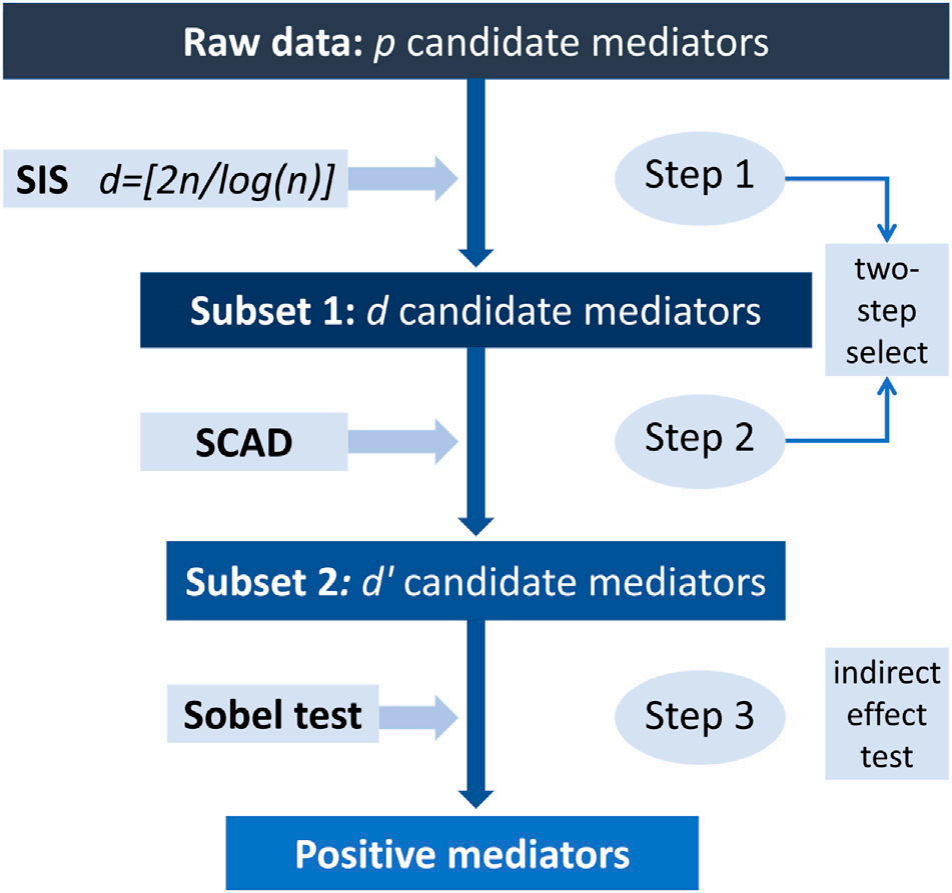
Overall workflow of the proposed procedure.

However, the maximum likelihood estimation is not feasible when *p* ≫ *n*. To identify the true-positive mediators, we consider the “two-step” method for dimension reduction. First, we apply SIS to reduce dimension from an ultrahigh level to a moderate one (Fan and Lv, 2008). Then we perform the regularization method with SCAD penalty for the SIS-selected subset. The Sobel test is applied to identify true mediators in SCAD-selected subset. Figure 2 shows the overall workflow of the proposed procedure. We will introduce details below.

Step 1. (*Screening*) Using SIS to reduce candidate mediators from *p* dimension to *d* dimension, we identify a subset *S*_1_ = {*M*_*k*_: 1 ≤ *k* ≤ *p*}. Here we select *d* = [2*n*/ log(*n*)] mediators instead of [*n*/ log(*n*)] recommended by Fan and Lv (2008) to contain more positive mediators in subset *S*_1_, because mediators are related to exposure and outcome simultaneously.

Step 2. (*SCAD-penalized selection*) Further selection with SCAD penalty for the subset $S_{2}=\left\{M_{k}:{\hat{\beta }}_{k}\neq 0\right\}$ based on *M*_*k*_ ∈ *S*_1_ is applied by minimizing the following objective function with penalty:

$$Q\left(\beta \right)=L\left(\beta \right)+\overset{p}{\underset{j=1}{\sum }}p_{\lambda }\left(\left|\beta_{j}\right|\right),$$

where *L*(***β***) has been shown in Eq. 6, and

$$p_{\lambda }^{\prime }\left(\left|\beta \right|\right)=\lambda I\left(|\beta |\leq \lambda \right)+\frac{{\left(a\lambda -|\beta |\right)}_{+}}{a-1}I\left(|\beta |>\lambda \right)\;a>2\;\lambda >0.$$

Here we choose the regularization parameters by 5-fold cross-validation. Gorst-Rasmussen and Scheike (2012) implemented the SCAD penalized method for additive hazards model in R package *ahaz*.

Step 3. (*Effect decomposition and IE test*) Referring to the single mediator (Lange and Hansen, 2011) and two mediators based on the additive hazards model (Huang and Yang, 2017), we use the counterfactual hazard difference to measure the effect difference when exposure changes from *x* to x*. Counterfactual hazard difference, also named total effect (TE), includes two parts: direct effect (DE) and IE. DE represents the exposure directly caused effect. And IE expresses the effect caused by exposure through mediators indirectly.

Defining $M_{k}^{x}$ and $M_{k}^{x^{*}}$ as the mediator value with different exposure value *x* and x* separately, we have the following decomposition of TE (for more details see Supplementary Appendix):

$$\begin{array}{ll} \text{TE}= & \lambda \left(T\left(x*,M_{1}\left(x*\right),\ldots ,M_{p}\left(x*\right)\right);t|Z\right)-\lambda \left(T\left(x,M_{1}\left(x\right),\ldots ,M_{p}\left(x\right)\right);t|Z\right)\\ = & \lambda \left(T\left(x*,M_{1}\left(x*\right),\ldots ,M_{p}\left(x*\right)\right);t|Z\right)-\lambda \left(T\left(x*,M_{1}\left(x\right),\ldots ,M_{p}\left(x\right)\right);t|Z\right)\\ & +\lambda \left(T\left(x*,M_{1}\left(x\right),\ldots ,M_{p}\left(x\right)\right);t|Z\right)-\lambda \left(T\left(x,M_{1}\left(x\right),\ldots ,M_{p}\left(x\right)\right);t|Z\right)\\ = & \gamma \left(x*-x\right)+\left(\alpha_{1}\beta_{1}+\cdots +\alpha_{p}\beta_{p}\right)\left(x*-x\right)\\ = & \text{DE}+\text{IE} \end{array}$$

Then we apply the Sobel mediation significance test to subset *S*_2_ to pick out true-positive mediators from candidates by significant IE. According to Sobel (1987), we have the null hypothesis *H*_0_: *α*_*k*_ *β*_*k*_ = 0 and following *p* value calculating formula:

$$\begin{array}{l} P_{raw,k}=2\left\{1-\phi \left(\frac{\left|\hat{\alpha_{k}}\hat{\beta_{k}}\right|}{{\hat{\sigma }}_{\alpha_{k}\beta_{k}}}\right)\right\}, \end{array}$$ (7)

where ${\hat{\sigma }}_{\alpha_{k}\beta_{k}}=\sqrt{{\hat{\alpha }}_{k}^{2}{\hat{\sigma }}_{\beta_{k}}^{2}+{\hat{\beta }}_{k}^{2}{\hat{\sigma }}_{\alpha_{k}}^{2}}$ is the estimated standard error, and ${\hat{\alpha }}_{k}$ is the estimator of *α*_*k*_, ${\hat{\beta }}_{k}$ is the estimator of *β*_*k*_, ${\hat{\sigma }}_{\alpha_{k}}^{2}$ is the estimated variance of *α*_*k*_, and ${\hat{\sigma }}_{\beta_{k}}^{2}$ is the estimated variance of *β*_*k*_.

## 3 Results

### 3.1 Simulation Studies

This section demonstrates the simulation results of the proposed procedure with high-dimensional mediator’s selection and IE estimation in a series of simulation studies.

#### 3.1.1 Simulation Design

We generate hazard rate of survival outcome based on additive hazards model $\lambda_{i}\left(t_{i}|X_{i},Z_{i},M_{i}\right)=5t+X_{i}+0.4Z_{1i}+0.4Z_{2i}+\sum_{k=1}^{p}\beta_{k}M_{ki}$ and high-dimensional mediators based on linear model *M*_*ki*_ = *c*_*k*_ + *α*_*k*_ *X*_*i*_ + 0.4*Z*_1*i*_ + 0.4*Z*_2*i*_ + *e*_*ki*_. The simulation data are generated according to the following parameter settings with different sample size (n = 500, 1,000) and mediator dimensions (*p* = 10,000, 20,000, 50,000, and 100,000). The censoring time follows the uniform distribution as *U*(0, *c*). By adjusting constant *c*, we control the censoring rate from 15% to 50% with a 5% gap to see the level of sensitivity of the proposed procedure with different censoring rates. For each scenario, we generate 500 replicates.

• *X*_*i*_ ∼ *B*(1, 0.6) is the exposure.
• *c*_*k*_ ∼ *U*(0, 0.5) is the intercept and *e*_*ki*_ ∼ *N*(0, 1) is the random error.
• $\alpha^{T}=\left(1,1,1,1,0.5,0.5,0,0,\underset{9992}{\underset{︸}{0,\ldots ,0}}\right)$ and $\beta^{T}=\left(1,1,1,1,0,0,0.5,0.5,\underset{9992}{\underset{︸}{0,\ldots ,0}}\right)$ .
• *Z*_*i*1_ ∼ *B*(1, 0.3), *Z*_*i*2_ ∼ *U*(0, 1).

Candidates with nonzero IEs are positive mediators, and zero IEs are negative mediators. We use TPR (true-positive rate), FP (false-positive number), and FDP to evaluate mediator’s selection. And we use estimated IE, coverage probability, estimated standard error, and empirical standard error to evaluate IE estimation. To control the multiple hypothesis test error, we apply the BH (Benjamini and Hochberg, 1995) method to adjust the estimated *p* value. However, the BH method assumes independent hypotheses, which are not satisfied in some cases. We also consider the BY (Benjamini and Yekutieli, 2001) method for dependent situations. We apply both BH method and BY method for adjusting to compare their performance under different scenarios.

#### 3.1.2 Simulation Results

**FIGURE 3:**
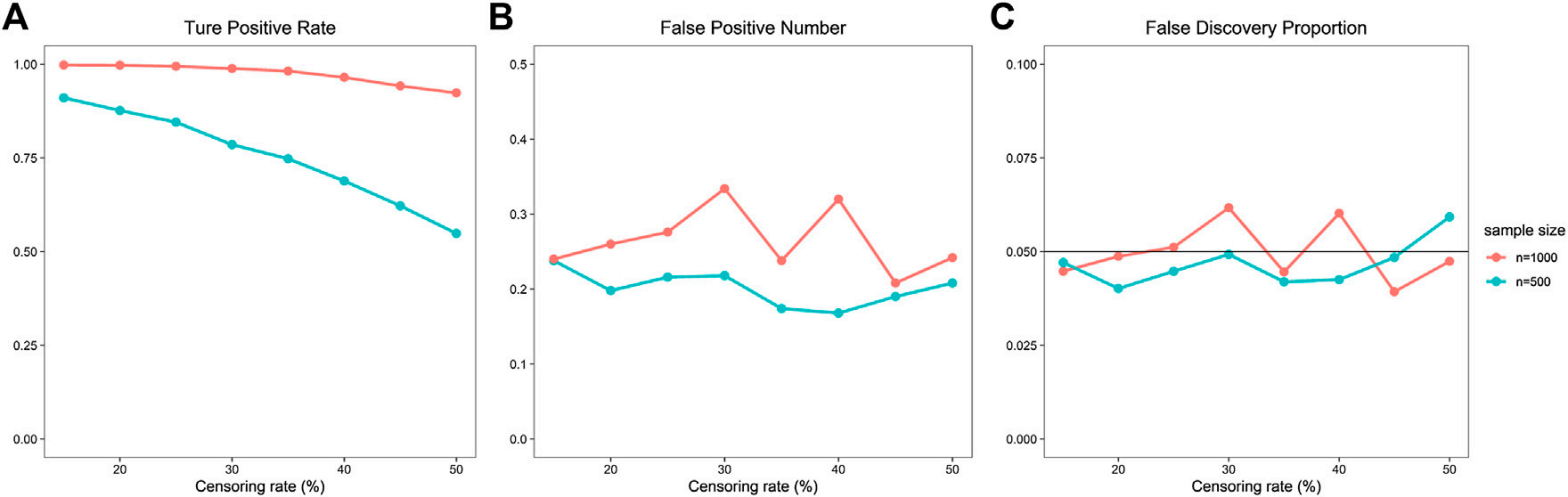
Select accuracy of the proposed procedure. **(A)** shows TPR variation of the proposed procedure with different censoring rate and sample size, **(B)** shows FP variation, **(C)** shows FDP variation.

**FIGURE 4:**
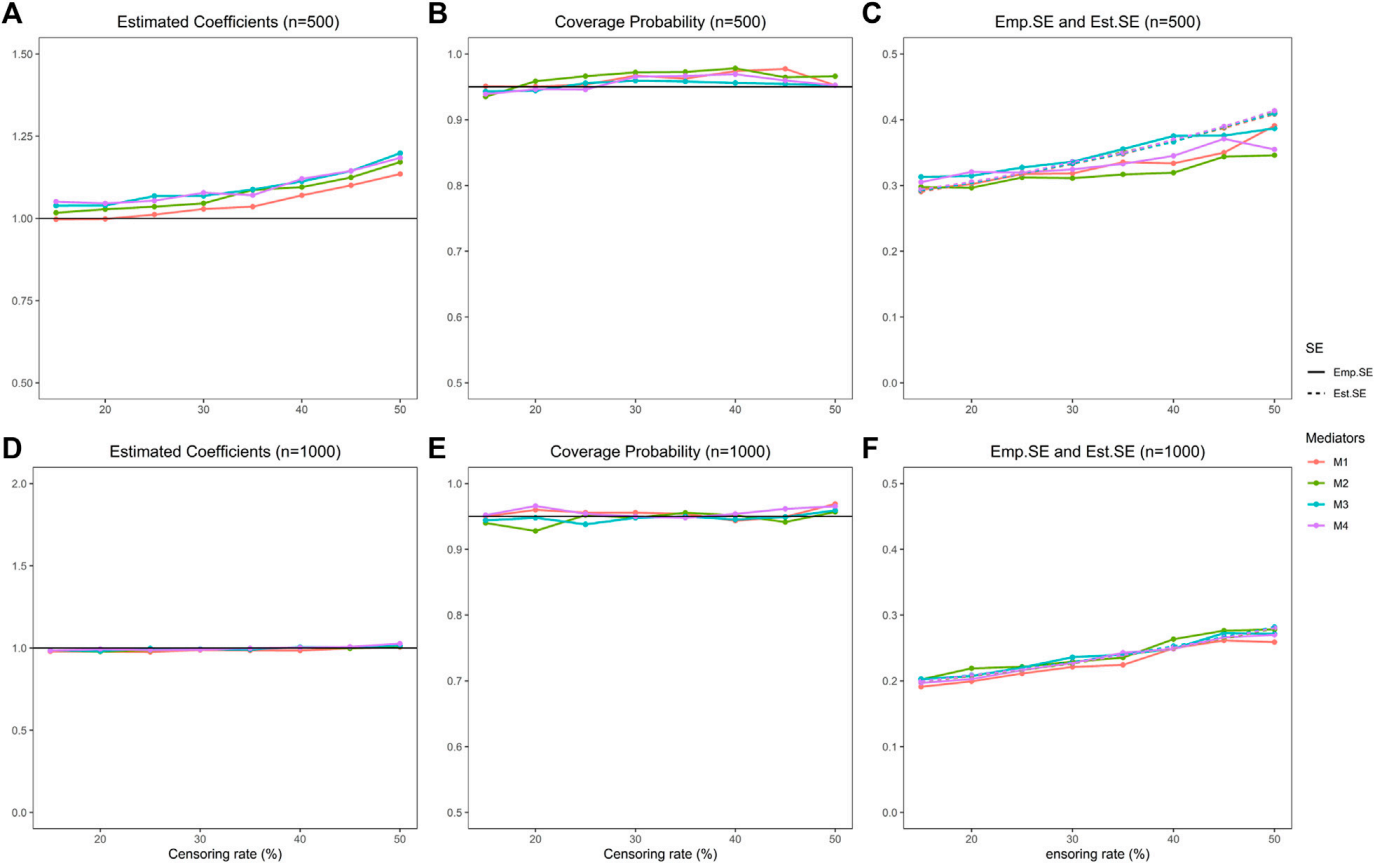
Indirect effect estimation of the proposed procedure. **(A)** is the estimated coefficients of four mediators with sample size 500 in simulation studies, **(B)** is the coverage probability of four mediators with sample size 500, **(C)** is the empirical standard error and estimated standard error of four mediators with sample size 500. **(D)**, **(E)**, **(F)** represent the same simulation results as **(A)**, **(B)**, **(C)** correspondingly with sample size 1000.

We demonstrate the proposed procedure’s performance with simulation results summarized in Tables 1, 2, visualized in Figures 3, 4. Figure 3 and Table 1 both show the accuracy of the mediator’s selection with censoring rates ranges from 15% to 50%, 10,000 mediators, and sample sizes of 500 and 1,000 respectively. In general, selection performs better in sample size 1,000 than 500, and the BH method (shown at the first line) performs better (higher TPR and acceptable FDP) than the BY method (shown at the second line). Considering the mediator’s independence assumption, we adopt the BH method into the proposed procedure. Under the adjustment of the BH method, the lowest TPR equals 0.5485 with sample size of 500 and censoring rate of 50%. TPR rises near 1 with the increase of sample size and decrease of censoring rate. The scenario with 1,000 samples and a 30% censoring rate has the highest FP (0.3340) and FDP (0.0617) simultaneously. The naive method estimates the IE for each mediator separately and applies multiple hypothesis adjustments to all candidate mediators without variable selection. Simulation results demonstrate the proposed procedure has better selection performance than the naive method.

**TABLE 1:** Select accuracy of the proposed procedure compared with naive method.

Censoring rate	Sample size	Proposed procedure			Naive method		
		TPR	FP	FDP	TPR	FP	FDP
15%	n = 500	0.9105	0.2380	0.0471	0.0830	< 0.001	< 0.001
		0.8345	0.0160	0.0038	0.0230	< 0.001	< 0.001
	n = 1,000	0.9980	0.2400	0.0447	0.7100	< 0.001	< 0.001
		0.9950	0.0200	0.0040	0.4735	< 0.001	< 0.001
20%	n = 500	0.8765	0.1980	0.0402	0.0645	< 0.001	< 0.001
		0.7915	0.0160	0.0036	0.0130	< 0.001	< 0.001
	n = 1,000	0.9975	0.2600	0.0488	0.6230	< 0.001	< 0.001
		0.9890	0.0360	0.0072	0.3815	< 0.001	< 0.001
25%	n = 500	0.8455	0.2160	0.0448	0.0410	< 0.001	< 0.001
		0.7290	0.0240	0.0061	0.0095	< 0.001	< 0.001
	n = 1,000	0.9945	0.2760	0.0512	0.5350	< 0.001	< 0.001
		0.9855	0.0200	0.0041	0.3005	< 0.001	< 0.001
30%	n = 500	0.7855	0.2180	0.0493	0.0280	< 0.001	< 0.001
		0.6550	0.0140	0.0036	0.0045	< 0.001	< 0.001
	n = 1,000	0.9885	0.3340	0.0617	0.4630	< 0.001	< 0.001
		0.9725	0.0220	0.0044	0.2210	< 0.001	< 0.001
35%	n = 500	0.7480	0.1740	0.0420	0.0240	< 0.001	< 0.001
		0.6115	0.0200	0.0059	0.0025	< 0.001	< 0.001
	n = 1,000	0.9820	0.2380	0.0446	0.3480	< 0.001	< 0.001
		0.9575	0.0200	0.0040	0.1560	< 0.001	< 0.001
40%	n = 500	0.6885	0.1680	0.0425	0.0120	< 0.001	< 0.001
		0.5475	0.0160	0.0060	0.0015	< 0.001	< 0.001
	n = 1,000	0.9650	0.3200	0.0602	0.2700	< 0.001	< 0.001
		0.9285	0.0180	0.0037	0.1110	< 0.001	< 0.001
45%	n = 500	0.6220	0.1900	0.0485	0.0055	< 0.001	< 0.001
		0.4655	0.0080	0.0034	0.0005	< 0.001	< 0.001
	n = 1,000	0.9420	0.2080	0.0393	0.2035	< 0.001	< 0.001
		0.8975	0.0200	0.0042	0.0705	< 0.001	< 0.001
50%	n = 500	0.5485	0.2080	0.0593	0.0055	< 0.001	< 0.001
		0.4145	0.0100	0.0050	0.0005	< 0.001	< 0.001
	n = 1,000	0.9235	0.2420	0.0474	0.1340	< 0.001	< 0.001
		0.8545	0.0140	0.0031	0.0465	< 0.001	< 0.001

Each scenario has two results, the first line represents the BH-adjusted *p* value, and the second line is the BY-adjusted *p* value; TPR, percentage of correctly selected positive mediators; FP number, number of incorrectly selected negative mediators; FDP, percentage of FP mediators among all selected. The results are an average of 500 replications.

**TABLE 2:** Indirect effect estimation of the proposed procedure.

Mediation	Estimation	cen = 15%		cen = 25%		cen = 35%		cen = 50%	
		n = 500	n = 1,000	n = 500	n = 1,000	n = 500	n = 1,000	n = 500	n = 1,000
	Est.	0.9973	0.9795	1.0114	0.9763	1.0355	0.9854	1.1351	1.0051
*M*_1_	CP	0.9509	0.9500	0.9534	0.9559	0.9626	0.9539	0.9524	0.9690
(1,1) = 1	Emp.SE	0.2937	0.1910	0.3175	0.2113	0.3354	0.2243	0.3909	0.2590
	Est.SE	0.2907	0.1997	0.3166	0.2174	0.3481	0.2389	0.4086	0.2806
	Est.	1.0171	0.9854	1.0355	0.9848	1.0866	0.9962	1.1713	1.0086
*M*_2_	CP	0.9351	0.9400	0.9662	0.9520	0.9727	0.9556	0.9661	0.9568
(1,1) = 1	Emp.SE	0.2978	0.2022	0.3123	0.2213	0.3169	0.2354	0.3460	0.2781
	Est.SE	0.2924	0.1991	0.3192	0.2167	0.3511	0.2384	0.4124	0.2796
	Est.	1.0387	0.9860	1.0678	0.9970	1.0877	0.9913	1.1984	1.0156
*M*_3_	CP	0.9430	0.9440	0.9556	0.9380	0.9581	0.9499	0.9523	0.9591
(1,1) = 1	Emp.SE	0.3131	0.2028	0.3275	0.2204	0.3554	0.2400	0.3868	0.2714
	Est.SE	0.2926	0.2003	0.3184	0.2186	0.3489	0.2395	0.4094	0.2816
	Est.	1.0510	0.9845	1.0539	0.9875	1.0706	0.9978	1.1842	1.0259
*M*_4_	CP	0.9390	0.9520	0.9459	0.9540	0.9667	0.9480	0.9522	0.9654
(1,1) = 1	Emp.SE	0.3051	0.1969	0.3198	0.2162	0.3329	0.2428	0.3547	0.2699
	Est.SE	0.2941	0.1995	0.3190	0.2174	0.3499	0.2390	0.4137	0.2805
	Est.	0.2354	0.0916	0.3143	0.1348	0.3465	0.1302	0.3417	0.1423
*M*_5_	CP	0.6071	0.3571	0.4231	0.3684	0.5333	0.3529	0.6500	0.5455
(0.5,0) = 0	Emp.SE	0.2106	0.2029	0.1076	0.1883	0.1281	0.2490	0.2653	0.2662
	Est.SE	0.1473	0.1000	0.1634	0.1091	0.1732	0.1240	0.2073	0.1389
	Est.	0.0985	0.1518	0.0599	0.2226	0.2380	0.2593	0.3769	0.2927
*M*_6_	CP	0.5263	0.7000	0.4706	0.3750	0.5263	0.2500	0.2308	0.3529
(0.5,0) = 0	Emp.SE	0.3193	0.1412	0.3669	0.0725	0.2928	0.0587	0.3874	0.0546
	Est.SE	0.1643	0.0988	0.1794	0.1043	0.1852	0.1196	0.2396	0.1451
	Est.	(—)	0.0097	(—)	0.0019	(—)	0.0647	(—)	−0.0012
*M*_7_	CP	(—)	0.3077	(—)	0.1667	(—)	0.2500	(—)	0.2000
(0,0.5) = 0	Emp.SE	(—)	0.1225	(—)	0.1347	(—)	0.1207	(—)	0.1623
	Est.SE	(—)	0.0554	(—)	0.0616	(—)	0.0636	(—)	0.0769
	Est.	0.0901	0.0772	0.0871	0.0802	0.0771	0.1376	0.0261	0.1526
*M*_8_	CP	0.8000	0.2500	0.5000	0.4000	0.7500	0.4286	1.0000	0.5000
(0,0.5) = 0	Emp.SE	0.1546	0.0944	0.1935	0.0952	0.1877	0.0145	0.1869	0.0155
	Est.SE	0.0941	0.0547	0.1071	0.0587	0.1162	0.0649	0.1217	0.0754

The first column represents *M*_*k*_(*α*, *β*), product of *αβ* is the real IE; cen, abbreviation of censoring rate; Est., the mean of coefficient estimation; CP, coverage probability, the proportion of replicates which 95% confidence interval (CI) cover the true value of the coefficient; Emp. SE, empirical standard error, calculated standard error from the estimation of all replicates; Est. SE, mean of estimated standard error among all replicates. (-) represents those mediators haven’t been selected among 500 replicates. The results are an average of 500 replications.

To verify the preponderance of the proposed procedure, we compare it with the joint method, the lasso method, and the Cox model method. The joint method uses the joint significant test in place of the Sobel test; meanwhile, the “two-step” variable selection is the same as the proposed procedure. The FP and FDP of the proposed procedure are much lower than the joint method. The comparison of the proposed procedure and the joint method is shown in Supplementary Table S1. The lasso method replaces the SCAD penalty with the lasso penalty in the regularization step. For both lasso- and SCAD-penalized selection, we apply 5-fold cross-validation to optimize the regularization parameters. The TPR of the proposed procedure is higher than the lasso method. The comparison of the proposed procedure and lasso method is accessible at Supplementary Table S2. The Cox model method fits the Cox proportion hazards model instead of the additive hazards model in regularization and IE estimation parts. In penalized step, we apply 5-fold cross-validation to optimize the regularization parameters for both Cox and additive hazards models. The TPR of the proposed procedure is higher than the Cox model method. The comparison of the proposed procedure and Cox model method is shown in Supplementary Table S3.

We also inspect the performance of the proposed procedure with more mediators like 20,000, 50,000, and 100,000. Under these circumstances, TPR hardly changes, whereas FP and FDP raise slowly with the increase of mediators dimension. Results of more mediators selected by the proposed procedure are available at Supplementary Table S4. To make simulations closer to the real world, we set the dependent mediators in another scenario. Results show that with the increase of mediator’s correlation, TPR decreases, and FP and FDP increase. The assumption of the BH method is not satisfied with dependent mediators. We pick the BY method to adjust the dependent *p* value. The dependent mediator’s variable selection results are available at Supplementary Table S5. We also look over the selection performance of the proposed procedure under four different coefficients, and the results are shown in Supplementary Table S6.

In addition, we evaluate the IE estimation performance. We show the results of 10,000 mediators and sample sizes 500 and 1,000 in Figure 4 and Table 2 (results of censoring rate equal to 15%, 25%, 35%, and 50% are in Table 2, and the rest shown in Supplementary Table S7). In summary, the estimation performs pretty well and improves with the increase of sample size. The estimated IE is close to the true value with a slight bias. The coverage probabilities are approximately 0.95. The estimated standard error and empirical standard error are close to each other.

In a word, the proposed procedure has good performance in high-dimensional mediation analysis based on the additive hazards model with high selected accuracy and exact estimation performance. Therefore, we apply it to the TCGA (The Cancer Genome Atlas) lung cancer data.

### 3.2 Application

Lung cancer is still the most fatal cancer worldwide with many pathogenic factors such as tobacco smoking and air pollution; 80% to 85% of lung cancers were caused by smoking (Wild et al., 2020). Nicotine in tobacco may result in genetic mutations. To find out whether smoking leads to lung cancer by affecting the DNA methylation, we applied the proposed procedure to the TCGA lung cancer cohort study involving DNA methylation data (907 samples measured by Illumina Infinium HumanMethylation450 platform), phenotype data (1,299 samples), and survival data (1,145 samples) for lung squamous cell carcinoma and lung adenocarcinoma. DNA methylation values recorded via BeadStudio software were continuous from 0 to 1 representing the intensity ratio. Thus, a higher value represents a higher degree of methylation, and so does the lower one.

After sample matching and data cleaning among the above data sets, we obtained 833 patients; 41.2% (343) were female, and 68.4% (570) were smokers. The patients’ ages ranged from 33 to 90 years with a median of 67 years. The overall survival time represented the days from first diagnosed to death or the last follow-up date. The median survival time was 652 days (1.79 years).

SIS based on the marginal correlation between tobacco smoking status and DNA methylation was first applied to reduce DNA methylation sites from 365,306 to 2*n*/log(*n*) (=248). Then we applied the SCAD penalty for a further dimension reduction and get a 25 sites subset. We applied the Sobel test and BH method to that subset for IE hypothesis testing. cg19757631, cg08636115, cg05147638, cg24720672, and cg08530838 are significant DNA methylation sites with adjusted *p* value < 0.05. We are interested in mediating DNA methylation markers, which increase lung cancer patients’ survival hazards. Therefore, we focus on the CpG sites with positive IEs ( $\hat{\alpha_{k}}\hat{\beta_{k}}\;>$ 0): cg19757631, cg08636115, cg05147638, and cg24720672.

Table 3 shows mediated CpG sites with positive IE. The estimated IE was represented by $\hat{\alpha }\hat{\beta }$ . The TE (effect between exposure and outcome with covariates) of tobacco smoking on lung cancer patients’ survival equaled 0.0137 (95% CI = −0.0252–0.0526), and its DE (effect between exposure and outcome adjusting for mediators and covariates) equals 0.0171 (95% CI = −0.0244–0.0585). The IEs of four significant mediated CpG sites cg19757631, cg08636115, cg05147638, and cg24720672 are equal 0.0296 (95% CI = 0.0129–0.0464), 0.0263 (95% CI = 0.0093–0.0433), 0.0185 (95% CI = 0.0047–0.0323), and 0.0269 (95% CI = 0.0100–0.0438), respectively.

**TABLE 3:** Significant mediate CpG sites with positive indirect effect.

	Est. IE	95% CI	P(BH)	P(BY)	SE	$\hat{\beta }$	$\hat{\alpha }$	Chr	Gene
cg19757631	0.0296	(0.0129–0.0464)	0.0112	0.0428	0.0086	−0.2806	−0.1056	chr1	SRM
cg08636115	0.0263	(0.0093–0.0433)	0.0152	0.0581	0.0087	−0.3811	−0.0690	chr1	PRDM16
cg05147638	0.0185	(0.0047–0.0323)	0.0422	0.1612	0.0070	0.4918	0.0376	chr12	COPZ1
cg24720672	0.0269	(0.0100–0.0438)	0.0151	0.0575	0.0086	−1.4889	−0.0181	chr15	LOC283663

Est. IE, the estimated IE ( $\hat{\alpha }\hat{\beta }$ ); P(BH), BH-adjusted *p* value; P(BY), BY-adjusted *p* value; SE, the estimated standard error; Chr, the chromosome where CpG is located in; Gene, the CpG located or nearest gene.

Bakulski et al. (2019) studied DNA methylation sites associated with smoking exposure in TCGA lung adenocarcinoma tissue samples and found cg19757631 is significant (FDR-adjusted *p* value < 0.05). In their study, the estimated methylation change of smokers versus never smokers is −12.28% (adjusted *p* value* = *4.81E-06), which is consistent with ours ( $\hat{\beta }=-0.2806$ ). The experiment results from Fei et al. (2019) about gene PRDM16 (cg08636115 located) suggest that PRDM16 is a metastasis suppressor and potential therapeutic target for lung adenocarcinomas, which has the same conclusion as ours $\left(\hat{\beta }=-0.3811\right)$ . Shtutman et al. (2011) explained the operational mechanism of COPZ1 (cg05147638 located) in the tumor cell: the function-based genomic screening identified COPZ1 gene is essential in different tumor cell types instead of normal cells. Gene COPZ1 methylation is harmful. This conclusion approves our results: $\hat{\beta }=$ 0.4918. As for CpG site cg24720672, we find some researches about leukemia—a kind of cancer, and we infer it has the similar mechanism in tumor tissue as lung cancer (Nair, 2016; Zhang et al., 2018; Jiang et al., 2020).

The real data application identifies four significant mediated DNA methylation sites with positive IEs between tobacco smoking and lung cancer patients’ survival. CpG site cg19757631 is a mediator having a known relationship with tobacco smoking (Bakulski et al., 2019). CpG sites cg05147638, cg08636115, and cg24720672 are newly addressed mediators. Besides, we also apply the naive method to the TCGA lung cancer data set, but nothing has been identified.

## 4 Discussion

High-dimensional data analysis methods are becoming increasingly important with the development of sequence technologies. Mediation analysis is effective for identifying potential pathways. High-dimensional mediation models provide a new tool for biomarker finding (e.g., identifying DNA methylation sites as the potential mediator between smoking and cancer patient’s survival). In this article, we propose an approach for high-dimensional mediation analysis based on the additive hazards model, which identifies true mediators and estimates IEs. We first use the “two-step” variable selection method (contains SIS and SCAD-penalized method) to reduce high-dimensional mediators. Then we apply the Sobel test and the BH method for multiple IE hypothesis testing. Besides, we also use the BY method, a more serious adjusting method for dependent multiple hypothesis, to see the results of unsuitable method (and the results demonstrate it does bring a lower TPR). Simulation studies show good performance of the proposed procedure. The real data application identifies four DNA methylation sites with positive IEs between smoking and lung cancer patient’s survival time. The proposed procedure and its application results are valuable theoretically and practically for high-dimensional mediation analysis based on the additive hazards model.

High-dimensional mediation analysis is still at the early stage and yet to be developed further. For example, the proposed procedure for mediation analysis assumes no unmeasured confounder effect. Potential confounders could affect the IE estimation in many observational studies. Methods to incorporate confounders in the high-dimensional mediation model using propensity score or other approaches are still under development. On the other hand, we consider high-dimensional mediation analysis for longitudinal or repeated-measures data. The IE estimation methods for correlated high-dimensional mediators are also of interest.

## Data Availability

The TCGA lung cancer data we used in the real data application can be found in (https://xenabrowser.net/) without limitation. The proposed procedure is implemented by R. The corresponding R code can be found at https://github.com/Cui-yd/HMA.
