# Supplementary material for: High-Dimensional Mediation Analysis Based on Additive Hazards Model for Survival Data
Source: Front Genet. 2021 Dec 23;12:771932. doi: 10.3389/fgene.2021.771932 (PMC8734376; doi:10.3389/fgene.2021.771932)
Supplement: Supplementary file 1 [file Table5.PDF]

frontiers

Supplementary Material

S5 TABLE.

Select accuracy of the proposed procedure with dependent mediators

censoring rate	δ	n=500			n=1000		
		TPR	FP	FDP	TPR	FP	FDP
15%	0	0.9105	0.2380	0.0471	0.9980	0.2400	0.0447
		0.8345	0.0160	0.0038	0.9950	0.0200	0.0040
	0.2	0.8290	0.3040	0.0612	0.9910	0.4240	0.0801
		0.7155	0.0200	0.0062	0.9825	0.1040	0.0209
	0.5	0.5255	0.4300	0.1211	0.8795	0.8080	0.1653
		0.3660	0.1340	0.0612	0.7765	0.4720	0.1095
	0.8	0.2135	0.2960	0.2090	0.4000	0.4620	0.1733
		0.1385	0.1740	0.1960	0.2565	0.2440	0.1512
25%	0	0.8455	0.2160	0.0448	0.9945	0.2760	0.0512
		0.7290	0.0240	0.0061	0.9855	0.0200	0.0041
	0.2	0.7300	0.2080	0.0493	0.9735	0.3800	0.0717
		0.5900	0.0080	0.0024	0.9455	0.0720	0.0147
	0.5	0.4190	0.2720	0.1040	0.7730	0.6860	0.1563
		0.2750	0.0840	0.0531	0.6335	0.3620	0.0971
	0.8	0.1745	0.2500	0.2219	0.2990	0.4100	0.2126
		0.1210	0.1460	0.1905	0.1890	0.2180	0.1859
35%	0	0.7480	0.1740	0.0420	0.9820	0.2380	0.0446
		0.6115	0.0200	0.0059	0.9575	0.0200	0.0040
	0.2	0.6415	0.1980	0.0496	0.9390	0.3320	0.0631
		0.4970	0.0240	0.0079	0.8830	0.0660	0.0143
	0.5	0.3525	0.2480	0.1167	0.6600	0.5740	0.1471
		0.2450	0.0860	0.0605	0.5150	0.2860	0.0935
	0.8	0.1570	0.2400	0.2343	0.2155	0.3140	0.2248
		0.1160	0.1560	0.2212	0.1450	0.1880	0.2240

δ represents for the correlation between mediators. $\tilde{M}_1 = M_1$, $\tilde{M}_k = M_k + \delta_k M_{k-1}$ for $k \geq 2$. Each scenario has two results, the first line represents the BH-adjusted p-value and the second line is the BY-adjusted p-value. TPR:true positive rate; FP: false positive number; FDP: false discovery proportion. The results are the average of 500 replications.
