# Supplementary material for: High-Dimensional Mediation Analysis Based on Additive Hazards Model for Survival Data
Source: Front Genet. 2021 Dec 23;12:771932. doi: 10.3389/fgene.2021.771932 (PMC8734376; doi:10.3389/fgene.2021.771932)
Supplement: Supplementary file 2 [file Table4.PDF]

Supplementary Material

S4 TABLE.

Select accuracy of the proposed procedure with more mediators

mediators dimension	censoring rate	15%		25%		35%	
	sample size	500	1000	500	1000	500	1000
p=10,000	TPR	0.9105	0.9980	0.8455	0.9945	0.7480	0.9820
		0.8345	0.9950	0.7290	0.9855	0.6115	0.9575
	FP	0.2380	0.2400	0.2160	0.2760	0.1740	0.2380
		0.0160	0.0200	0.0240	0.0200	0.0200	0.0200
	FDP	0.0471	0.0447	0.0448	0.0512	0.0420	0.0446
		0.0038	0.0040	0.0061	0.0041	0.0059	0.0040
p=20,000	TPR	0.9110	0.9990	0.8573	0.9945	0.7635	0.9820
		0.8175	0.9960	0.7528	0.9855	0.6419	0.9575
	FP	0.4700	0.5060	0.3785	0.2760	0.4324	0.2380
		0.0180	0.0440	0.0226	0.0200	0.0270	0.0200
	FDP	0.0855	0.0914	0.0732	0.0512	0.0869	0.0446
		0.0038	0.0086	0.0052	0.0041	0.0063	0.0040
p=50,000	TPR	0.9135	0.9970	0.8000	0.9955	0.7500	0.9845
		0.8305	0.9945	0.7125	0.9825	0.6786	0.9535
	FP	0.7480	0.9480	0.7000	0.9680	0.2857	1.0500
		0.0340	0.0840	0.0500	0.0680	0.0000	0.0540
	FDP	0.1357	0.1555	0.1219	0.1571	0.0643	0.1685
		0.0077	0.0162	0.0167	0.0137	0.0000	0.0106
p=100,000	TPR	0.9135	0.9970	0.8000	0.9955	0.7500	0.9845
		0.8305	0.9945	0.7125	0.9825	0.6786	0.9535
	FP	0.7480	0.9480	0.7000	0.9680	0.2857	1.0500
		0.0340	0.0840	0.0500	0.0680	0.0000	0.0540
	FDP	0.1357	0.1555	0.1219	0.1571	0.0643	0.1685
		0.0077	0.0162	0.0167	0.0137	0.0000	0.0106

Each scenario has two results, the first line represents the BH-adjusted p-value and the second line is the BY-adjusted p-value. TPR:true positive rate; FP: false positive number; FDP: false discovery proportion. The results are the average of 500 replications.
