# Supplementary material for: High-Dimensional Mediation Analysis Based on Additive Hazards Model for Survival Data
Source: Front Genet. 2021 Dec 23;12:771932. doi: 10.3389/fgene.2021.771932 (PMC8734376; doi:10.3389/fgene.2021.771932)
Supplement: Supplementary file 3 [file Table1.PDF]

Supplementary Material

S1 TABLE.

Select accuracy of proposed procedure compared with joint significant test method

censoring rate	sample size	proposed procedure			joint test method		
		TPR	FP	FDP	TPR	FP	FDP
15%	n=500	0.9105	0.2380	0.0471	0.9340	2.5040	0.3333
		0.8345	0.0160	0.0038	0.8735	1.2820	0.2285
	n=1000	0.9980	0.2400	0.0447	0.9985	2.1240	0.2663
		0.9950	0.0200	0.0040	0.9950	0.9160	0.1538
20%	n=500	0.8765	0.1980	0.0402	0.9180	2.4140	0.3289
		0.7915	0.0160	0.0036	0.8370	1.2400	0.2287
	n=1000	0.9975	0.2600	0.0488	0.9985	2.3820	0.2965
		0.9890	0.0360	0.0072	0.9950	1.0020	0.1665
25%	n=500	0.8455	0.2160	0.0448	0.8905	2.5560	0.3507
		0.7290	0.0240	0.0061	0.7910	1.3580	0.2503
	n=1000	0.9945	0.2760	0.0512	0.9970	2.6820	0.3096
		0.9855	0.0200	0.0041	0.9910	1.0380	0.1749
30%	n=500	0.7855	0.2180	0.0493	0.8510	2.6180	0.3746
		0.6550	0.0140	0.0036	0.7215	1.2240	0.2503
	n=1000	0.9885	0.3340	0.0617	0.9925	2.7580	0.3284
		0.9725	0.0220	0.0044	0.9820	1.1120	0.1885
35%	n=500	0.7480	0.1740	0.0420	0.8190	2.4800	0.3658
		0.6115	0.0200	0.0059	0.6860	1.2300	0.2605
	n=1000	0.9820	0.2380	0.0446	0.9890	3.0060	0.3428
		0.9575	0.0200	0.0040	0.9715	1.1060	0.1853
40%	n=500	0.6885	0.1680	0.0425	0.7615	2.4220	0.3844
		0.5475	0.0160	0.0060	0.6295	1.1360	0.2536
	n=1000	0.9650	0.3200	0.0602	0.9800	3.0540	0.3540
		0.9285	0.0180	0.0037	0.9520	1.1600	0.2011
45%	n=500	0.6220	0.1900	0.0485	0.7020	2.3800	0.4100
		0.4655	0.0080	0.0034	0.5535	1.0840	0.2682
	n=1000	0.9420	0.2080	0.0393	0.9640	3.2600	0.3652
		0.8975	0.0200	0.0042	0.9215	1.0860	0.1902
50%	n=500	0.5485	0.2080	0.0593	0.6250	2.4460	0.4587
		0.4145	0.0100	0.0050	0.4895	1.1500	0.3172
	n=1000	0.9235	0.2420	0.0474	0.9525	3.3840	0.3796
		0.8545	0.0140	0.0031	0.8960	1.1540	0.2064

In joint test method, the first two steps are the same as the proposed procedure, while the indirect effect examination method in the third step is joint method instead of Sobel method. Each scenario has two results, the first line represents the BH-adjusted p-value and the second line is the BY-adjusted p-value. TPR:true positive rate; FP: false positive number; FDP: false discovery proportion. The results are the average of 500 replications.
