# Supplementary material for: High-Dimensional Mediation Analysis Based on Additive Hazards Model for Survival Data
Source: Front Genet. 2021 Dec 23;12:771932. doi: 10.3389/fgene.2021.771932 (PMC8734376; doi:10.3389/fgene.2021.771932)
Supplement: Supplementary file 4 [file Table2.PDF]

Supplementary Material

S2 TABLE.

Select accuracy of proposed procedure compared with lasso regularization method

censoring rate	sample size	proposed procedure			lasso method		
		TPR	FP	FDP	TPR	FP	FDP
15%	n=500	0.9105	0.2380	0.0471	0.7980	0.0520	0.0128
		0.8345	0.0160	0.0038	0.6725	0.0060	0.0016
	n=1000	0.9980	0.2400	0.0447	0.9895	0.0380	0.0077
		0.9950	0.0200	0.0040	0.9775	0.0040	0.0008
20%	n=500	0.8765	0.1980	0.0402	0.7450	0.0660	0.0176
		0.7915	0.0160	0.0036	0.6175	0.0040	0.0010
	n=1000	0.9975	0.2600	0.0488	0.9785	0.0580	0.0118
		0.9890	0.0360	0.0072	0.9580	0.0120	0.0024
25%	n=500	0.8455	0.2160	0.0448	0.6825	0.0740	0.0220
		0.7290	0.0240	0.0061	0.5620	0.0160	0.0065
	n=1000	0.9945	0.2760	0.0512	0.9725	0.0400	0.0080
		0.9855	0.0200	0.0041	0.9425	0.0020	0.0004
30%	n=500	0.7855	0.2180	0.0493	0.6375	0.0760	0.0229
		0.6550	0.0140	0.0036	0.5195	0.0080	0.0033
	n=1000	0.9885	0.3340	0.0617	0.9580	0.0580	0.0116
		0.9725	0.0220	0.0044	0.9165	0.0100	0.0021
35%	n=500	0.7480	0.1740	0.0420	0.5705	0.0900	0.0337
		0.6115	0.0200	0.0059	0.4415	0.0140	0.0095
	n=1000	0.9820	0.2380	0.0446	0.9375	0.0580	0.0122
		0.9575	0.0200	0.0040	0.8815	0.0020	0.0004
40%	n=500	0.6885	0.1680	0.0425	0.4935	0.0560	0.0244
		0.5475	0.0160	0.0060	0.3890	0.0080	0.0061
	n=1000	0.9650	0.3200	0.0602	0.9035	0.0660	0.0136
		0.9285	0.0180	0.0037	0.8185	0.0080	0.0018
45%	n=500	0.6220	0.1900	0.0485	0.4150	0.1060	0.0557
		0.4655	0.0080	0.0034	0.3200	0.0160	0.0201
	n=1000	0.9420	0.2080	0.0393	0.8610	0.0600	0.0136
		0.8975	0.0200	0.0042	0.7760	0.0140	0.0050
50%	n=500	0.5485	0.2080	0.0593	0.3635	0.1200	0.0624
		0.4145	0.0100	0.0050	0.2865	0.0180	0.0198
	n=1000	0.9235	0.2420	0.0474	0.8075	0.0500	0.0127
		0.8545	0.0140	0.0031	0.7070	0.0060	0.0019

In lasso method, the first step and the third step are the same as proposed procedure, while the regularization penalty in the second step is lasso instead of SCAD. Each scenario has two results, the first line represents the BH-adjusted p-value and the second line is the BY-adjusted p-value. TPR:true positive rate; FP: false positive number; FDP: false discovery proportion. The results are the average of 500 replications.
