# Supplementary material for: High-Dimensional Mediation Analysis Based on Additive Hazards Model for Survival Data
Source: Front Genet. 2021 Dec 23;12:771932. doi: 10.3389/fgene.2021.771932 (PMC8734376; doi:10.3389/fgene.2021.771932)
Supplement: Supplementary file 5 [file Table6.pdf]

Supplementary Material

S6 TABLE.

Select accuracy of the proposed procedure with different coefficients

Coef. set	Sample size	cen=15%			cen=25%			cen=35%		
		TPR	FP	FDP	TPR	FP	FDP	TPR	FP	FDP
Set 1	n=500	0.9105	0.2380	0.0471	0.8455	0.2160	0.0448	0.7480	0.1740	0.0420
		0.8345	0.0160	0.0038	0.7290	0.0240	0.0061	0.6115	0.0200	0.0059
	n=1000	0.9980	0.2400	0.0447	0.9945	0.2760	0.0512	0.9820	0.2380	0.0446
		0.9950	0.0200	0.0040	0.9855	0.0200	0.0041	0.9575	0.0200	0.0040
Set 2	n=500	0.9620	0.2180	0.0422	0.9140	0.2480	0.0463	0.8400	0.1740	0.0367
		0.9140	0.0040	0.0008	0.8415	0.0080	0.0019	0.7255	0.0060	0.0030
	n=1000	1.0000	0.2060	0.0385	0.9985	0.2140	0.0411	0.9930	0.2120	0.0405
		0.9995	0.0140	0.0028	0.9935	0.0100	0.0020	0.9840	0.0080	0.0016
Set 3	n=500	0.9365	0.3260	0.0618	0.8425	0.2900	0.0589	0.7090	0.2260	0.0509
		0.8775	0.0280	0.0077	0.7490	0.0220	0.0065	0.5730	0.0400	0.0305
	n=1000	0.9980	0.1900	0.0350	0.9910	0.2460	0.0460	0.9625	0.2800	0.0535
		0.9955	0.0440	0.0085	0.9825	0.0260	0.0052	0.9310	0.0380	0.0085
Set 4	n=500	0.8480	0.2620	0.0522	0.7705	0.2140	0.0496	0.6410	0.2260	0.0592
		0.7445	0.0280	0.0069	0.6370	0.0360	0.0238	0.4955	0.1000	0.0864
	n=1000	0.9945	0.3020	0.0562	0.9820	0.2820	0.0529	0.9505	0.2340	0.0454
		0.9830	0.0420	0.0084	0.9585	0.0440	0.0088	0.9020	0.0240	0.0051

Set 1: $\alpha^T = (1, 1, 1, 1, 0.5, 0.5, 0, 0, \underbrace{0, \ldots, 0}_{9992})$, $\beta^T = (1, 1, 1, 1, 0, 0, 0.5, 0.5, \underbrace{0, \ldots, 0}_{9992})$

Set 2: $\alpha^T = (1, 1, 1, 1, 0, 0, 0, 0, \underbrace{0, \ldots, 0}_{9992})$, $\beta^T = (1, 1, 1, 1, 0, 0, 0, 0, \underbrace{0, \ldots, 0}_{9992})$

Set 3: $\alpha^T = (1.56, 1.87, 1.32, 1.95, 0.73, 0.62, 0, 0, \underbrace{0, \ldots, 0}_{9992})$, $\beta^T = (1.68, 1.59, 1.74, 1.48, 0, 0, 0.65, 0.68, \underbrace{0, \ldots, 0}_{9992})$

Set 4: $\alpha^T = (1, 1, 1, 1, 1, 1, 0, 0, \underbrace{0, \ldots, 0}_{9992})$, $\beta^T = (1, 1, 1, 1, 0, 0, 1, 1, \underbrace{0, \ldots, 0}_{9992})$

Each scenario has two results, the first line represents the BH-adjusted p-value and the second line is the BY-adjusted p-value. cen: abbreviation of censoring rate; TPR:true positive rate; FP: false positive number; FDP: false discovery proportion. The results are the average of 500 replications.
