# Supplementary material for: High-Dimensional Mediation Analysis Based on Additive Hazards Model for Survival Data
Source: Front Genet. 2021 Dec 23;12:771932. doi: 10.3389/fgene.2021.771932 (PMC8734376; doi:10.3389/fgene.2021.771932)
Supplement: Supplementary file 6 [file Table3.PDF]

Supplementary Material

S3 TABLE.

Select accuracy of proposed procedure compared with Cox method

censoring rate	sample size	proposed procedure			Cox model method		
		TPR	FP	FDP	TPR	FP	FDP
15%	n=500	0.9105	0.2380	0.0471	0.8280	0.1080	0.0263
		0.8345	0.0160	0.0038	0.7345	0.0200	0.0114
	n=1000	0.9980	0.2400	0.0447	0.9920	0.0660	0.0128
		0.9950	0.0200	0.0040	0.9780	0.0040	0.0008
20%	n=500	0.8765	0.1980	0.0402	0.7670	0.1340	0.0362
		0.7915	0.0160	0.0036	0.6810	0.0360	0.0293
	n=1000	0.9975	0.2600	0.0488	0.9820	0.1080	0.0207
		0.9890	0.0360	0.0072	0.9675	0.0120	0.0024
25%	n=500	0.8455	0.2160	0.0448	0.7045	0.1540	0.0438
		0.7290	0.0240	0.0061	0.6250	0.0480	0.0362
	n=1000	0.9945	0.2760	0.0512	0.9755	0.0660	0.0132
		0.9855	0.0200	0.0041	0.9515	0.0020	0.0004
30%	n=500	0.7855	0.2180	0.0493	0.6595	0.1460	0.0460
		0.6550	0.0140	0.0036	0.5580	0.0720	0.0594
	n=1000	0.9885	0.3340	0.0617	0.9635	0.0880	0.0175
		0.9725	0.0220	0.0044	0.9235	0.0120	0.0041
35%	n=500	0.7480	0.1740	0.0420	0.5840	0.1760	0.0650
		0.6115	0.0200	0.0059	0.4985	0.0820	0.0728
	n=1000	0.9820	0.2380	0.0446	0.9390	0.0920	0.0186
		0.9575	0.0200	0.0040	0.8925	0.0080	0.0019
40%	n=500	0.6885	0.1680	0.0425	0.5245	0.1320	0.0532
		0.5475	0.0160	0.0060	0.4485	0.0760	0.0806
	n=1000	0.9650	0.3200	0.0602	0.9060	0.1040	0.0213
		0.9285	0.0180	0.0037	0.8370	0.0100	0.0020
45%	n=500	0.6220	0.1900	0.0485	0.4595	0.1840	0.0907
		0.4655	0.0080	0.0034	0.3710	0.1160	0.1251
	n=1000	0.9420	0.2080	0.0393	0.8530	0.0920	0.0203
		0.8975	0.0200	0.0042	0.7900	0.0240	0.0105
50%	n=500	0.5485	0.2080	0.0593	0.4105	0.1760	0.0852
		0.4145	0.0100	0.0050	0.3220	0.1360	0.1460
	n=1000	0.9235	0.2420	0.0474	0.8035	0.0900	0.0263
		0.8545	0.0140	0.0031	0.7195	0.0300	0.0232

In Cox model method, the first step is same as the proposed procedure, while the regularization step and the indirect effect examination step are fit by Cox proportion hazards model instead of additive hazards model. Each scenario has two results, the first line represents the BH-adjusted p-value and the second line is the BY-adjusted p-value. TPR:true positive rate; FP: false positive number; FDP: false discovery proportion. The results are the average of 500 replications.
