# Supplementary material for: High-Dimensional Mediation Analysis Based on Additive Hazards Model for Survival Data
Source: Front Genet. 2021 Dec 23;12:771932. doi: 10.3389/fgene.2021.771932 (PMC8734376; doi:10.3389/fgene.2021.771932)
Supplement: Supplementary file 7 [file DataSheet1.PDF]

Supplementary Material

S1 APPENDIX.

The high-dimensional mediation model based on additive hazards model has the form as:

$$\lambda_i(t|X_i, M_i, Z_i) = \lambda_{0i}(t) + \gamma X_i(t) + \boldsymbol{\theta}^T \mathbf{Z}_i(t) + \boldsymbol{\beta}^T \mathbf{M}_i(t) \qquad i = 1, 2, \dots, n, \tag{S1}$$

$$M_{ki} = c_k + \alpha_k X_i(t) + \boldsymbol{\vartheta}^T \mathbf{Z}_i(t) + e_{ki} \qquad k = 1, 2, \dots, p \tag{S2}$$

With the assumption of consistency, we have the corresponding assumptions about above model:

(A 1.) $X \perp T(x, m_1, m_2, \dots, m_p)|Z$; there is no confounding for the effect between X and T conditional on Z.

(A 2.) For any $k = 1, 2, \dots, p$, $M_k \perp T(x, m_1, m_2, \dots, m_p)|X, Z$; there is no confounding for the effect between M_k and T conditional on X and Z.

(A 3.) For any $k = 1, 2, \dots, p$, $X \perp M_k|Z$; there is no confounding for the effect between X and M_k conditional on Z.

(A 4.) For any $k = 1, 2, \dots, p$, $M_k^{x^*} \perp T(x, m_1, m_2, \dots, m_p)|Z$; there is no X induced factor confound the pathway from M to T conditional on Z, where x^* is intervention for X with different value than x.

Referring to Huang and Yang (2017), they have studied the direct and indirect effect of two mediators with Aalen additive hazards model under the counterfactual framework, we expand this decomposition to high-dimensional scenario. With the four assumptions, the cumulative distribution function of the counterfactual survival time can be expressed as a multiple integral considering of the distributions of p mediators:

$$\begin{aligned}
&F_{T(x,M_1(x^*),\dots,M_p(x^*))}(t|Z) \\
&= \int \dots \int F_{T(x,m_1,\dots,m_p)}(t|Z, M_1(x^*) = m_1, \dots, M_p(x^*) = m_p) \mathrm{d}F_{M_1}(m_1|Z, m_1) \dots \\
&\qquad \mathrm{d}F_{m_p}(M_p|Z, m_p) \\
&= \int \dots \int F_{T(x,m_1,\dots,m_p)}(t|Z) \mathrm{d}F_{M_1}(m_1|Z) \dots \mathrm{d}F_{M_p}(m_p|Z) && (A4) \\
&= \int \dots \int F_{T(x,m_1,\dots,m_p)}(t|Z) \mathrm{d}F_{M_1}(m_1|Z, x^*) \dots \mathrm{d}F_{M_p}(m_p|Z, x^*) && (A3) \\
&= \int \dots \int F_{T(x,m_1,\dots,m_p)}(t|Z, x) \mathrm{d}F_{M_1}(m_1|Z, x^*) \dots \mathrm{d}F_{M_p}(m_p|Z, x^*) && (A1) \\
&= \int \dots \int F_{T(x,m_1,\dots,m_p)}(t|Z, x, m_1, \dots, m_p) \mathrm{d}F_{M_1}(m_1|Z, x^*) \dots \mathrm{d}F_{M_p}(m_p|Z, x^*) && (A2) \\
&= \int \dots \int F_T(t|Z, x, m_1, \dots, m_p) \mathrm{d}F_{M_1}(m_1|Z, x^*) \dots \mathrm{d}F_{M_p}(m_p|Z, x^*).
\end{aligned}$$

In this way, the probability density function can be expressed as:

$$
\begin{aligned}
& f_{T(x,m_1,...,m_p)}(t|Z) \\
=& \mathrm{d}F_{T(x,m_1,...,m_p)}(t|Z) \\
=& \int ... \int \mathrm{d}F_T(t|Z,x,m_1,...,m_p)\mathrm{d}F_{M_1}(m_1|Z,x^*)...\mathrm{d}F_{M_p}(m_p|Z,x^*) \\
=& \int ... \int f_T(t|Z,x,m_1,...,m_p)\mathrm{d}F_{M_1}(m_1|Z,x^*)...\mathrm{d}F_{M_p}(m_p|Z,x^*).
\end{aligned}
$$

We propose a linearly predicted survival model with high-dimensional mediators based on Eq. S1 as follow:

$$\lambda_i = \lambda_0(t) + \theta^T Z + \gamma X + W_{\beta i}, \tag{S3}$$

where $W_{\beta i} = \beta_1 M_{1i} ... + \beta_2 M_{2i}$, $W_{\beta i}$ is a function of x^* and following a normal distribution G_{w_β} : $W_\beta(x^*) \sim N(\mu_{w_\beta}, \sigma^2_{W_\beta})$, where $\mu_{w_\beta} = \beta_1(c_1 + \vartheta^T Z + \alpha_1 x^*) + ... + \beta_p(c_p + \vartheta^T Z + \alpha_p x^*)$, $\sigma^2_{W_\beta} = \beta_1^2\sigma^2_{M_1} + ... + \beta_p^2\sigma^2_{M_p}$.

With four assumptions mentioned at beginning and the results derived above, the counterfactual outcome defined as hazard can be deduced as follows:

$$
\begin{aligned}
& \lambda(T(x,M_1,...,M_p;t|Z) \\
=& \frac{\int f_{T(x,M_1(x^*),...,M_p(x^*))}(t|Z)}{1 - \int F_{T(x,M_1(x^*),...,M_p(x^*))}(t|Z)} \\
=& \frac{\int f_T(t|x)\mathrm{d}G_{W_\beta}(x^*)}{1 - \int F_T(t|x)\mathrm{d}G_{W_\beta}(x^*)} \\
=& \frac{\int \lambda(t|x)e^{-\lambda(t|x)}\mathrm{d}G_{W_\beta}(x^*)}{\int e^{-\lambda(t|x)}\mathrm{d}G_{W_\beta}(x^*)} \\
=& \mu_{W_\beta} + \lambda_0(t) + \theta^T Z + \gamma x - \sigma^2_{W_\beta} \\
=& \lambda_0(t) + \theta^T Z + \gamma x + \beta_1(c_1 + \vartheta^T Z + \alpha_1 x^*) + ... + \beta_p(c_p + \vartheta^T Z + \alpha_p x^*) - \sigma^2_{W_\beta} \\
=& \{\lambda_0(t) + \theta^T Z + (\beta_1 c_1 + ... + \beta_p c_p + \beta_1\vartheta^T Z + ... + \beta_p\vartheta^T Z) - (\beta_1^2\sigma^2_{M_1} + ... + \beta_p^2\sigma^2_{M_p})\} \\
& + \gamma x + \beta_1\alpha_1 x^* + ... + \beta_p\alpha_p x^*.
\end{aligned}
$$

The third last equality utilizes the property of moment generating function of normal random variable since W_β is normally distributed.

By the definition of path-specific effects of high-dimensional mediators based on counterfactual frameworks, we can express the total causal effect on hazard rate as follows:

$$
\begin{aligned}
TE =& \lambda(T(x^*, M_1(x^*), \ldots, M_p(x^*)); t|Z) - \lambda(T(x, M_1(x), \ldots, M_p(x)); t|Z) \\
=& \lambda(T(x^*, M_1(x^*), \ldots, M_p(x^*)); t|Z) - \lambda(T(x^*, M_1(x), \ldots, M_p(x)); t|Z) \\
&+ \lambda(T(x^*, M_1(x), \ldots, M_p(x)); t|Z) - \lambda(T(x, M_1(x), \ldots, M_p(x)); t|Z) \\
=& \gamma(x^* - x) + (\alpha_1\beta_1 + \cdots + \alpha_p\beta_p)(x^* - x) \\
=& DE + IE,
\end{aligned}
$$

where TE denote the total effect; DE denote the direct effect; IE denote the indirect effect. We can also get the path-specific effects (IE_k) for each mediator M_k.

REFERENCES

Huang, Y.-T. and Yang, H.-I. (2017). Causal mediation analysis of survival outcome with multiple mediators. *Epidemiology (Cambridge, Mass.)* 28, 370
