# Supplementary material for: High-Dimensional Mediation Analysis Based on Additive Hazards Model for Survival Data
Source: Front Genet. 2021 Dec 23;12:771932. doi: 10.3389/fgene.2021.771932 (PMC8734376; doi:10.3389/fgene.2021.771932)
Supplement: Supplementary file 8 [file Table7.pdf]

Supplementary Material

S7 TABLE.

Performance of estimation with proposed procedure

mediation	estimation	cen=20%		cen=30%		cen=40%		cen=45%	
		n=500	n=1000	n=500	n=1000	n=500	n=1000	n=500	n=1000
	Est.	0.9983	0.9789	1.0285	0.9877	1.0700	0.9845	1.1004	0.9982
M_1	CP	0.9504	0.9600	0.9670	0.9558	0.9739	0.9433	0.9774	0.9495
(1,1)=1	Emp.SE	0.3020	0.1992	0.3183	0.2212	0.3337	0.2496	0.3500	0.2614
	Est.SE	0.3029	0.2082	0.3328	0.2272	0.3673	0.2499	0.3873	0.2653
	Est.	1.0278	0.9785	1.0460	0.9927	1.0949	1.0035	1.1246	0.9974
M_2	CP	0.9586	0.9280	0.9720	0.9479	0.9782	0.9517	0.9645	0.9415
(1,1)=1	Emp.SE	0.2966	0.2191	0.3110	0.2291	0.3194	0.2633	0.3438	0.2763
	Est.SE	0.3042	0.2074	0.3338	0.2265	0.3674	0.2504	0.3885	0.2644
	Est.	1.0394	0.9806	1.0686	0.9927	1.1125	1.0050	1.1442	1.0057
M_3	CP	0.9443	0.9480	0.9593	0.9479	0.9559	0.9455	0.9544	0.9492
(1,1)=1	Emp.SE	0.3146	0.2073	0.3362	0.2359	0.3753	0.2487	0.3760	0.2728
	Est.SE	0.3038	0.2087	0.3335	0.2285	0.3665	0.2526	0.3892	0.2667
	Est.	1.0457	0.9932	1.0781	0.9908	1.1203	1.0023	1.1445	1.0066
M_4	CP	0.9463	0.9660	0.9652	0.9500	0.9692	0.9538	0.9597	0.9615
(1,1)=1	Emp.SE	0.3207	0.2027	0.3245	0.2271	0.3451	0.2486	0.3710	0.2662
	Est.SE	0.3056	0.2083	0.3358	0.2273	0.3693	0.2508	0.3897	0.2656
	Est.	0.2657	0.1463	0.2341	0.1655	0.1683	0.0681	0.3715	0.0939
M_5	CP	0.5556	0.2143	0.4194	0.3500	0.5000	0.3750	0.3571	0.2105
(0.5,0)=0	Emp.SE	0.1615	0.1959	0.2979	0.2108	0.3850	0.2834	0.2858	0.3270
	Est.SE	0.1594	0.1034	0.1729	0.1188	0.1942	0.1279	0.2069	0.1463
	Est.	0.0710	0.1885	0.2184	0.2268	0.1457	0.2221	0.2384	0.2407
M_6	CP	0.5385	0.3889	0.4783	0.4286	0.4500	0.2353	0.2667	0.2941
(0.5,0)=0	Emp.SE	0.2997	0.1331	0.3054	0.0632	0.3969	0.1279	0.3859	0.1799
	Est.SE	0.1618	0.1057	0.1862	0.1165	0.1973	0.1225	0.2150	0.1339
	Est.	(-)	0.0003	(-)	0.0173	(-)	0.0051	(-)	0.0121
M_7	CP	(-)	0.2857	(-)	0.3333	(-)	0.3000	(-)	0.5000
(0,0.5)=0	Emp.SE	(-)	0.1211	(-)	0.1355	(-)	0.1518	(-)	0.1540
	Est.SE	(-)	0.0566	(-)	0.0629	(-)	0.0697	(-)	0.0710
	Est.	0.0935	0.0805	-0.0079	0.1105	0.0754	0.1088	-0.2972	0.1377
M_8	CP	0.8000	0.2222	0.4000	0.2857	0.7500	0.3750	0.0000	0.5000
(0,0.5)=0	Emp.SE	0.1307	0.0982	0.2103	0.0957	0.2068	0.0895	0.0000	0.0301
	Est.SE	0.0942	0.0583	0.1059	0.0648	0.1175	0.0662	0.1451	0.0690

The first column represents $M_k(\alpha, \beta)$, product of $\alpha\beta$ is the real IE. cen, abbreviation of censoring rate; Est., the mean of coefficient estimation; CP, coverage probability, the proportion of replicates which 95% confidence interval (CI) cover the true value of the coefficient; Emp. SE, empirical standard error, calculated standard error from the estimation of all replicates; Est. SE, mean of estimated standard error among all replicates. (-) represents those mediators haven't been selected among 500 replicates. The results are an average of 500 replications.
